# Supplementary material for: Identification of photoperiod-induced specific miRNAs in the adrenal glands of Sunite sheep (Ovis aries)
Source: Front Vet Sci. 2022 Jul 22;9:888207. doi: 10.3389/fvets.2022.888207 (PMC9354845; doi:10.3389/fvets.2022.888207)
Supplement: Supplementary file 1 [file Data_Sheet_1.ZIP › Supplementary file/miRDeep2 file.zip!/miRDeep2 file/Novel_Structure/SP42A1/Novel_36.pdf]

Provisional ID	:	NC_019484.2_26673
Score total	:	7.7
Score for star read(s)	:	3.9
Score for read counts	:	0.1
Score for mfe	:	2.1
Score for randfold	:	1.6
Score for cons. seed	:	
Total read count	:	12
Mature read count	:	11
Loop read count	:	0
Star read count	:	1

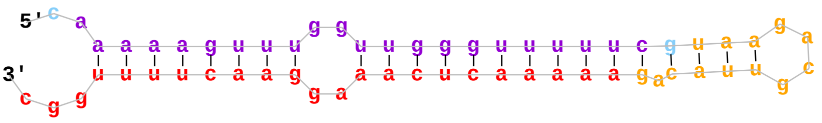

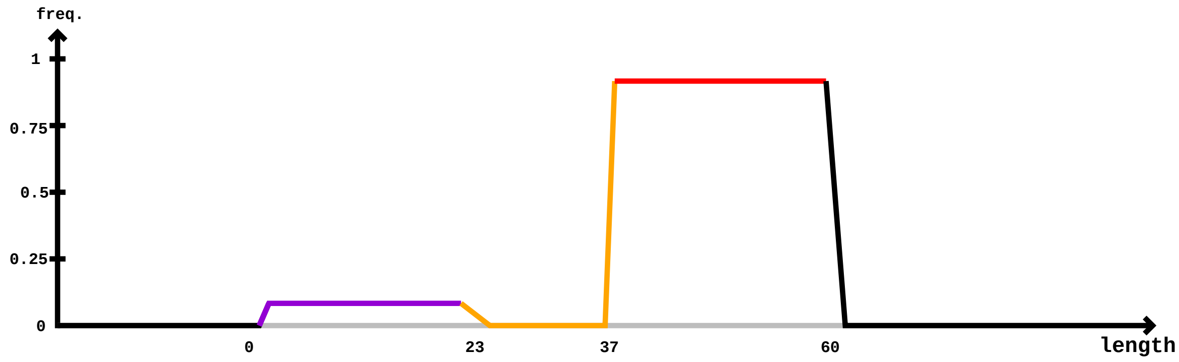

Star **Mature**

```
5'- uauaaauuauuggguuggccaaaaaguuugguuggguuuuucguaagacguuacagaaaaacucaaaggaacuuuuggccagcccaauauuauaugauuccaguuauacgaa   -3'    obs
    uauaaauuauuggguuggccaaaaaguuugguuggguuuuucguaagacguuacagaaaaacucaaaggaacuuuuggccagcccaauauuauaugauuccaguuauacgaa          exp
    .......(((((((((((((((((.((((..(((((((((((((((....)))).)))))))))))..)))))))))))))))))))))...((((((....))))))....   reads    mm      sample
    ....................aaaaaguuugguuggguuuuuc......................................................................   1        0       seq
    ........................................................aaaaacucaaaUgaacuuuuggc.................................   10       1       seq
    ........................................................aaaaacucaaaCgaacuuuuggc.................................   1        1       seq
```
